# Mulberry Fruit Prevents Diabetes and Diabetic Dementia by Regulation of Blood Glucose through Upregulation of Antioxidative Activities and CREB/BDNF Pathway in Alloxan-Induced Diabetic Mice

**DOI:** 10.1155/2020/1298691

**Published:** 2020-05-04

**Authors:** A Young Min, Jae-Myung Yoo, Dai-Eun Sok, Mee Ree Kim

**Affiliations:** ^1^Department of Food and Nutrition, Chungnam National University, Daejeon 34134, Republic of Korea; ^2^Korean Medicine-Application Center, Korea Institute of Oriental Medicine, Daegu 41062, Republic of Korea; ^3^Korean Medicine R&D Team 1, National Institute for Korean Medicine Development, Gyeongsan 38540, Republic of Korea; ^4^College of Pharmacy, Chungnam National University, Daejeon 34134, Republic of Korea

## Abstract

Although mulberry fruit has various beneficial effects, its effect on diabetes-related dementia remains unknown. We investigated whether the ethyl acetate fraction of ethanolic extract of mulberry fruit (MFE) could alleviate biochemical and behavioral deficits in alloxan-induced diabetic mice. In the diabetic mice, MFE considerably abolished multiple deficits, e.g., body weight reduction; water and food intake increase; and hyperglycemia, hyperlipidemia, hypoinsulinism, and hypertrophy of the liver, kidneys, spleen, and brain. A 200 mg/kg MFE dose reduced malondialdehyde levels and improved antioxidant enzyme activity in the liver, kidney, and brain tissues. MFE attenuated hyperglycemia-induced memory impairments and acetylcholine deprivation, protected neuronal cells in CA1 and CA3 regions via p-CREB/BDNF pathway activation, and reduced amyloid-*β* precursor protein and p-Tau expressions in the brain tissue. In conclusion, MFE exerts antidiabetic and neuroprotective effects by upregulating antioxidative activities and p-CREB/BDNF pathway in chronic diabetes. Therefore, MFE may be used as a therapeutic agent for diabetes and diabetic neurodegenerative diseases.

## 1. Introduction

The prevalence of diabetes, a chronic metabolic disorder characterized by persistent hyperglycemia, has been consistently increasing over the past few decades, becoming a global epidemic in modern society [1]. The progression of diabetes causes various complications, such as hypertension, hyperglycemia, hyperlipidemia, renal disorder, vascular diseases, and neurodegeneration [2]. Neurodegeneration is recognized as a cause of cognitive impairment observed in diabetic individuals [3]. Therefore, controlling hyperglycemia in patients with diabetes is important for preventing complications. Oxidative stress is the principal mechanism of many diabetic complications because of its active role in cellular injury in both neuronal and vascular cells [4]. A hyperglycemic state reduces antioxidant levels, consequently increasing free radical production [5]. Neurons are especially vulnerable to oxidative stress, and oxidative stress-induced mitochondrial damage leads to cell death [6]. A second possible mechanism is Tau protein, which is one of several proteins implicated in neurodegeneration. Tau protein is hyperphosphorylated in diabetic mouse models and may also underlie neuronal death [7].

Brain-derived neurotrophic factor (BDNF) in neuronal cells protects against oxidative stress and activates proliferation and plasticity in the hippocampus [8]. Furthermore, decreased BDNF expression in the brain tissue of humans with Alzheimer's disease and the animal models of the disorder has been reported [9]. Therefore, regulation of reactive oxygen species (ROS) generation and protective effects of BDNF in the brain are essential for the prevention/treatment of neurodegenerative diseases.

*Morus alba L*. (mulberry tree) of the Moraceae family has been widely cultivated in the world, and different parts of the plant are used as herbal medicine in East Asia [10]. Particularly, the mulberry tree's fruit, which can be consumed as food, is known to contain various phenolic compounds [11, 12] and other phytochemicals [13]. Mulberry fruit exhibits beneficial biological activities, such as antioxidant [14], antidiabetic [14], antiallergic [13], and neuroprotective effects [15]. These effects are believed to be caused by the combined actions of phytochemicals instead of a single compound in mulberry fruit. However, the effect of mulberry fruit on diabetic dementia remains unknown.

The purpose of this research was to explore whether the ethyl acetate fraction of ethanolic extract of mulberry fruit (MFE) prevents diabetic complications, such as memory deficits, in alloxan-induced diabetic mice [16], and its possible mechanisms of action. In this study, various diabetic biomarkers and antioxidant activities in multiple organs were evaluated for the antidiabetic effects of MFE. To examine the effect of MFE on diabetic dementia, behavioral tests and biomarkers of brain function were observed. Finally, the histological analysis of organs and the expression of BDNF, phosphorylated cAMP response element-binding protein (p-CREB), amyloid-*β* precursor protein (APP), and phosphorylated Tau (p-Tau) in hippocampal tissues were analyzed by immunoblot analysis to elucidate antidiabetic and neuroprotective actions of MFE.

## 2. Materials and Methods

### 2.1. Reagents

1X PBS and 1X TBS were purchased from Welgene, Inc. (Gyeongsan, Gyeongbuk, Korea). Specific antibodies against p-CREB, *β*-actin, and a horseradish peroxidase-conjugated IgG secondary antibody were procured from Cell Signaling Technology (Danvers, MA, USA). Specific antibodies against BDNF and insulin were purchased from Santa Cruz Biotechnology (Dallas, TX, USA). Specific antibodies against APP, Tau, and p-Tau were obtained from Abcam (Cambridge, UK). Radioimmunoprecipitation assay buffer was obtained from Merck Millipore (Darmstadt, Germany). Protease and phosphatase inhibitor cocktails were purchased from Roche Diagnostics (Indianapolis, IN, USA). CAS-Block™, ProLong™ Gold Antifade Mountant with DAPI, and goat anti-Rabbit IgG (H+L) Cross-Adsorbed Secondary Antibody (Alexa Fluor 488) were procured from Thermo Scientific (Waltham, MA, USA). A BCA protein assay kit was purchased from iNtRON Biotechnology, Inc. (Seongnam, Korea). Alloxan monohydrate and all other compounds detailed in the following sections were obtained from Sigma-Aldrich (St. Louis, MO, USA). All other chemicals were of analytical grade.

### 2.2. Preparation of MFE

MFE was prepared according to a previously reported method with some modifications [13]. Lyophilized mulberry fruit was obtained from S&D, Inc. (Yeongi, Korea) and then identified by Dr. Eun Soo Doh, a professor at the Department of Oriental Pharmaceutical Science, Joongbu University (Geumsan-gun, Korea). Briefly, lyophilized mulberry fruit (400 g) was extracted using 80% ethanol (1 L) in a bath sonicator for 1 d, and the mixture was then filtered using Whatman filter paper (No. 3). The process was repeated two more times. The whole filtrate was concentrated using a rotary vacuum evaporator (MG-2100, Buchi, Switzerland). The concentrate was added to ethyl acetate (1 : 1, *v*/*v*); then the layer of ethyl acetate was separated and evaporated. The dried residue of ethyl acetate extract (40 g) was dissolved in 1X PBS for an *in vivo* study.

### 2.3. Ultraperformance Liquid Chromatography-Tandem Mass Spectrometry Analysis

Ultraperformance liquid chromatography-tandem mass spectrometry analysis for the identification of phytochemicals in MFE was performed using a previously reported method [11].

### 2.4. Animals

Five-week-old male ICR mice, known as Swiss CD-1 mice [17], each weighing 25–30 g, were procured from Raon Bio Inc. (Yongin, Korea). Mice were housed in cages (5 mice per cage) under specific pathogen-free conditions (21–24°C and 40–60% relative humidity) with a 12 h light/dark cycle and provided free access to standard rodent food (OrientBio Inc., Sungnam, Korea) and water. All animal experiments were approved by the Committee of Animal Care and Experiment of Chungnam National University, Korea (CNU-00454), and performed according to the guidelines of the Animal Care and Use Committee at Chungnam National University.

### 2.5. Alloxan-Induced Diabetes

Alloxan-induced diabetes was performed using a modified version of previously reported method [18]. After acclimatization, mice were fasted for 8 h, and they were intravenously administered with or without alloxan solution (50 mg/kg). After 3 days, blood glucose levels of fasted mice were determined using a blood glucose monitoring meter (One Touch Ultra, LifeScan, Inc., Milpitas, USA). The next day, the diabetic mice (blood glucose ≥ 240 mg/dL) were administered with MFE (100 or 200 mg/kg orally) or glibenclamide (5 mg/kg orally) [19] once a day for 12 weeks. Each group included 10 mice. Food and water intake were monitored once daily, and body weight and blood glucose levels were monitored once weekly during the experiment.

### 2.6. Oral Glucose Tolerance Test

The oral glucose tolerance test was evaluated as previously reported [20]. Blood glucose levels were monitored using a blood glucose monitoring meter every 30 min over 2 h following the oral administration of a glucose solution (1 g/kg) in fasted mice. Mice were then sacrificed.

### 2.7. Determination of Biochemical Parameters and Organ Weights

On the final day, whole blood and organs were collected from anesthetized mice. To determine the levels of hemoglobin A1c (HbA1c), aspartate aminotransferase (AST), alanine aminotransferase (ALT), total cholesterol, triacylglycerol, HDL, blood urea nitrogen (BUN), uric acid (UA), creatinine, and C-reactive protein (CRP) in plasma, the collected blood was centrifuged (3,000 × *g* at 4°C) for 15 min; then the plasma was stored at −80°C until use. The plasma was analyzed using an automated chemistry analyzer (AU 5400, OLYMPUS, Shinjuku-ku, Tokyo, Japan) according to the manufacturer's instructions. The weights of isolated organs from the sacrificed mice were measured using a microbalance (TE214S, Sartorius, Goettingen, Germany).

### 2.8. Enzyme-Linked Immunosorbent Assay of Insulin

The amount of insulin in murine plasma was determined using ELISA kits (Morinaga Institute of Biological Science, Inc., Yokohama, Japan) according to the manufacturer's instructions.

### 2.9. Measurements of Lipid Peroxidation, Glutathione, and Antioxidant Enzyme Activities

Lipid peroxidation was determined using a previously reported method [21]. To measure the activities of superoxide dismutase (SOD) and glutathione reductase (GR) in the organs, liver, kidney, and brain tissues were homogenized in 20 mM phosphate buffer containing 0.1 M KCl, 1 mM EDTA, and 0.5% Triton X-100 (pH 7.4) using a homogenizer (EYELA MDC-2NS, Tokyo Rikakikai Co., Ltd., Tokyo, Japan). The homogenates were then centrifuged (17,000 × *g* at 4°C) for 30 min. Glutathione levels in the supernatants were analyzed using a Quantichrom Glutathione Assay Kit obtained from BioAssay Systems (Hayward, CA, USA) according to the manufacturer's protocol. For the SOD activity assay, the supernatant was mixed with an enzyme reaction buffer (50 mM potassium phosphate buffer containing 1 mM xanthine, 0.2 mM cytochrome, 50 mM potassium cyanide, and 0.1 mM EDTA); then xanthine oxidase was added into the mixed solution. The absorbance was measured at 550 nm using a microplate reader (DU650, Beckman Coulter, Brea, CA, USA). For the GR activity assay, the supernatant was mixed with 0.1 M phosphate buffer (1 M glutathione disulfide, 5 mM NADPH, and 0.5 mM EDTA, pH 7.0). The absorbance of the solution at 340 nm was monitored using a spectrophotometer.

### 2.10. Histological Analysis

Histological analysis was conducted following a modified previously published method [22]. To examine histological changes in the kidneys, pancreas, and brain, these tissues were fixed with 4% paraformaldehyde solution, made into paraffin blocks, and sectioned using a microtome. The sectioned tissues were deparaffinized, then incubated with 3% hydrogen peroxide in methanol for 5 min. The deparaffinized tissue slices were stained with hematoxylin-eosin or incubated with a 1 : 100 dilution of specific antibodies against insulin at 4°C overnight. For immunofluorescence staining, the tissue slices were incubated with anti-mouse/rabbit antibodies conjugated with fluorophore for 2 h in the dark. Finally, all stained tissue slices were embedded using mounting solution. Histological tissue changes in alloxan-induced diabetic mice were observed under a light microscope with 200x magnification or a fluorescence microscope with 100x magnification.

### 2.11. Behavior Tests

#### 2.11.1. Morris Water Maze Test

The Morris water maze test was performed using a previously reported method [23]. MFE was administered to alloxan-induced diabetic mice 1 h before the trial. 11 weeks after the MFE administration, the mice were subjected to the Morris water maze test for 6 days. On the first day, the mice were given swimming training for 120 s in the absence of the platform. Next, they were given four trial sessions per day with the platform for 4 days. The time interval between trial sessions was 20 min. When a mouse located the platform, it was permitted to remain on it for 10 s. If the mouse did not locate the platform within 120 s, it was placed on the platform for 10 s. On the final day, the mice were subjected to a probe trial, in which the platform was removed from the water pool, and they were allowed to swim for 120 s to search for the platform. The escape latency time, the time taken to cross the platform for the first time, and the number of crossing platform area in 120 s were recorded using a video camera (TGCAM-2000STA, Sambo Electronic Co., Ltd., Korea) connected to the EyeLine Video system.

#### 2.11.2. Passive Avoidance Test

The passive avoidance test was performed using previously reported method [23]. MFE was administered 1 h before the acquisition trial. For the learning trial, mice were placed in the illuminated compartment and the door between the two compartments was opened 20 s later. The time taken for the mouse to enter the dark compartment is defined as the latency time. When a mouse enters the dark compartment, the door was closed and an electrical shock (0.5 mA for 3 s) was delivered to the feet of the mouse through the stainless steel rods. If the mouse returns to the illuminated compartment, the mouse escapes the electrical shock. The latency time for entering the dark compartment was recorded up to 300 s. If a mouse did not enter the dark compartment up to 300 s, the latency time of the mouse was recorded as 300 s.

### 2.12. Determination of Acetylcholinesterase and Choline Acetyltransferase Activity

Acetylcholinesterase (AChE) activity was determined using a previously reported method [24]. Brain tissue was homogenized in ice-cold 1X PBS, then centrifuged (17,000 × *g* at 4°C) for 10 min. A supernatant (200 *μ*L) was mixed with 0.1 M phosphate buffer (15 mM acetylcholine iodide and 3 mM 5,5′-dithio-bis-2-nitobenzoic acid). The absorbance of the solution was measured at 412 nm using a microplate reader. Choline acetyltransferase (ChAT) activity in the supernatant was measured using an ELISA kit obtained from Nanjing Jiancheng Bioengineering Institute (Nanjing, China).

### 2.13. Immunoblot Analysis

Immunoblot analysis was evaluated using previously published method [23]. PVDF membranes that included blotted proteins were visualized using the WEST One™ western blot detection system (iNtRON Biotechnology, Inc., Seongnam, Korea). The level of target proteins was compared with that of a loading control (*β*-actin or the respective nonphosphorylated proteins), and the results were expressed as a ratio of density of each protein identified by a protein standard size marker (iNtRON Biotechnology, Inc., Seongnam, Korea). The relative density of the protein expression was quantitated by Matrox Inspector software (version 2.1 for Windows; Matrox Electronic Systems Ltd., Dorval, Quebec, Canada).

### 2.14. Statistical Analysis

The experimental results are reported as the mean ± SEM. One-way and two-way analysis of variances (ANOVAs) were used for multiple comparisons (GraphPad Prism version 5.03 for Windows, San Diego, CA, USA). Significant effects between treated groups were analyzed using Dunnett's test and the Newman–Keuls test for one-way ANOVAs and Bonferroni's post hoc test for two-way ANOVA. Differences at ^∗^*p* < 0.05 and ^∗∗^*p* < 0.01 levels were considered statistically significant.

## 3. Results

### 3.1. Antidiabetic Effects of MFE on Alloxan-Induced Diabetic Mice

**Figure 1:**
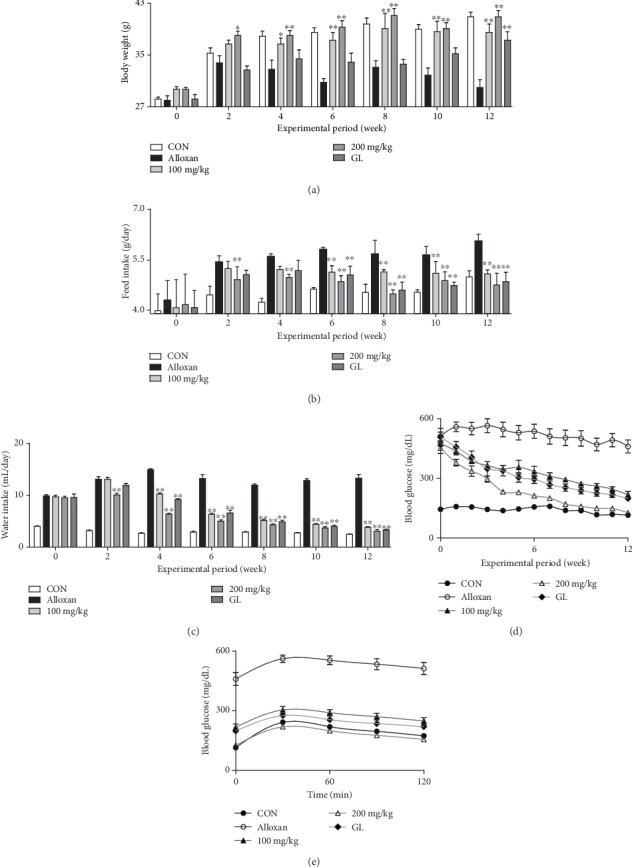
Antidiabetic effects of MFE on alloxan-induced diabetic mice. Mice were administered with MFE (0, 100, and 200 mg/kg orally) or glibenclamide (5 mg/kg orally) once a day for 12 weeks after an alloxan challenge (50 mg/kg). Body weight, blood glucose levels, and water and food intake were monitored once per week during the experimental period. In the oral glucose tolerance test, fasted mice were administered with a glucose solution (1 g/kg orally) before they were sacrificed. Data are expressed as the mean ± SEM. ^∗^*p* < 0.05 and ^∗∗^*p* < 0.01 versus the alloxan-treated group. (a) Body weight. (b) Food intake. (c) Water intake. (d) Blood glucose. (e) Oral glucose tolerance.

To investigate the effects of MFE on diabetes, changes in body weight and water and food intake were monitored in alloxan-induced diabetic mice. Loss of body weight and increased water and food intake are consistently observed in rodents exposed to alloxan [16]. After alloxan administration, body weight did not increase over the 3 to 12 weeks following treatment, although it did initially increase over the first 2 weeks (Figure 1(a)). Meanwhile, intake of water and food consistently increased (Figures 1(b) and 1(c)). MFE restored weight gain to levels similar to those of the control group. In addition, MFE normalized food intake until 12 weeks (Figure 1(b)), and 200 mg/kg MFE reduced water intake from 1 to 12 weeks after alloxan exposure (Figure 1(c)). Moreover, MFE-treated mice exhibited a gradual reduction in blood glucose levels, whereas mice treated with alloxan-alone maintained considerably high blood glucose levels (Figure 1(d)). Animals that received 200 mg/kg MFE showed normalized blood glucose levels from 8 to 12 weeks (Figure 1(d)). In the oral glucose tolerance test, MFE-treated mice had blood glucose levels that mirrored the pattern of the control group mice (Figure 1(e)). MFE showed better results than glibenclamide (5 mg/kg). These results suggest that MFE possesses antidiabetic properties. Moreover, preclinical antidiabetic effects of MFE surpassed those of the clinically used drug, glibenclamide.

### 3.2. Hypolipidemic and Antioxidant Effects of MFE in Alloxan-Induced Diabetic Mice

**Figure 2:**
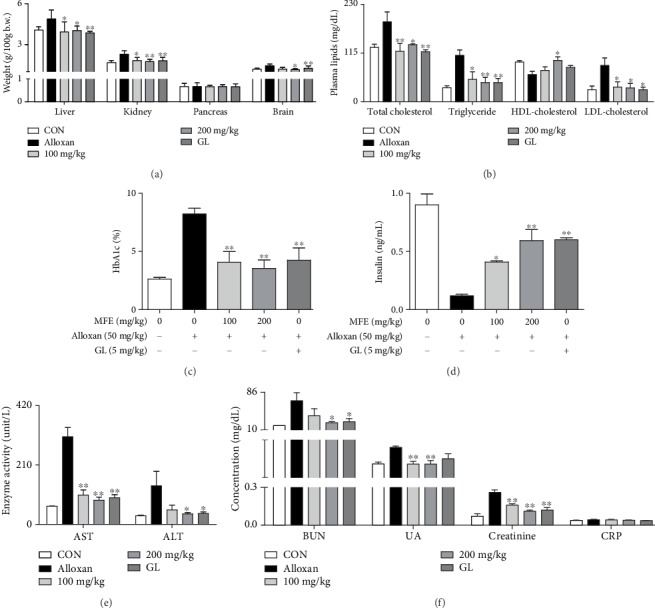
Effects of MFE on the organs of alloxan-treated mice. Liver, kidney, pancreas, and brain weights were measured after diabetic mice were sacrificed. Whole blood was collected from the anesthetized mice and centrifuged. HbA1c, triglyceride, total cholesterol, HDL, LDL, insulin, AST, ALT, BUN, UA, creatinine, and CRP levels were determined. Data are expressed as the mean ± SEM. ^∗^*p* < 0.05 and ^∗∗^*p* < 0.01 versus the alloxan-treated group. (a) Organ weights. (b) Blood lipid content. (c) HbA1c. (d) Insulin. (e) AST and ALT. (f) BUN, UA, creatinine, and CRP.

**Figure 3:**
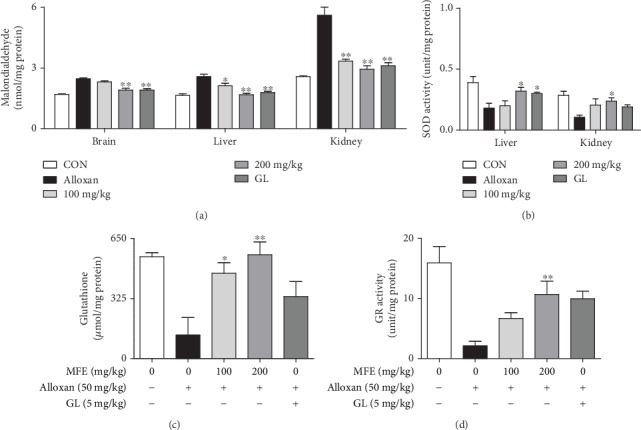
Effects of MFE on lipid peroxidation and antioxidant enzyme activity in the liver, kidney, and brain of alloxan-induced diabetic mice. Liver, kidney, and brain tissues isolated from the sacrificed mice were homogenized and centrifuged. Lipid peroxidation, glutathione, and SOD and GR activity were determined. Data are expressed as the mean ± SEM. ^∗^*p* < 0.05 and ^∗∗^*p* < 0.01 versus the alloxan-treated group. (a) Malondialdehyde. (b) SOD activity. (c) Glutathione. (d) GR activity.

**Figure 4:**
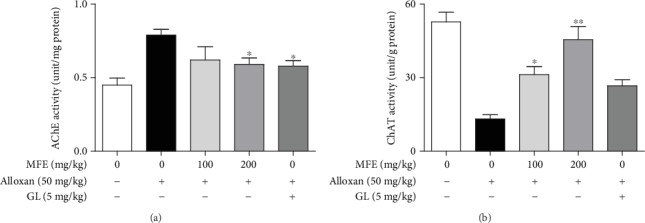
Effects of MFE on AChE and ChAT activity in the brain. The brains isolated from the sacrificed mice were homogenized and centrifuged. AChE and ChAT activities were determined. Data are expressed as the mean ± SEM values. ^∗^*p* < 0.05 and ^∗∗^*p* < 0.01 versus the alloxan-treated group. (a) AChE activity; (b) ChAT activity.

Because of the presence of antidiabetic effects of MFE, its effects on organ weight, lipid contents, and lipid peroxidation in alloxan-induced diabetic mice were further investigated. Diabetes is associated with hyperlipidemia [25], and hyperglycemia generates ROS throughout the body [26]. As shown in Figure 2(a), alloxan treatment increased liver, kidney, and brain weights, whereas it did not affect the weight of the pancreas in mice. In contrast, MFE significantly reduced liver, kidney, and brain weights. In addition, MFE suppressed HbA1c (Figure 2(c)), total cholesterol, triglyceride, and LDL levels (Figure 2(b)) and enhanced HDL (Figure 2(b)) and insulin (Figure 2(d)) levels. MFE also significantly inhibited the formation of the nephrotoxic markers, such as UA, BUN, creatinine, and CRP (Figure 2(f)) and the hepatotoxic markers, such as AST and ALT (Figure 2(e)). Furthermore, MFE diminished lipid peroxidation in liver, kidney, and brain tissues (Figure 3(a)) and increased the SOD activity in the livers and kidneys (Figure 3(b)), glutathione levels (Figure 3(c)), and GR activity (Figure 3(d)) in the brains. Interestingly, MFE inhibited AChE activity and increased ChAT activity in the brains of alloxan-induced diabetic mice (Figure 4). These findings indicate that MFE protects the brain and glucose-metabolizing organs, such as the liver, kidneys, and pancreas, through the upregulation of antioxidant enzymes. These effects of MFE might ameliorate diabetes by contributing to blood glucose homeostasis.

### 3.3. Protective Effects of MFE against Hyperglycemia-Induced Oxidative Stress in the Kidneys, Pancreas, and Brain

**Figure 5:**
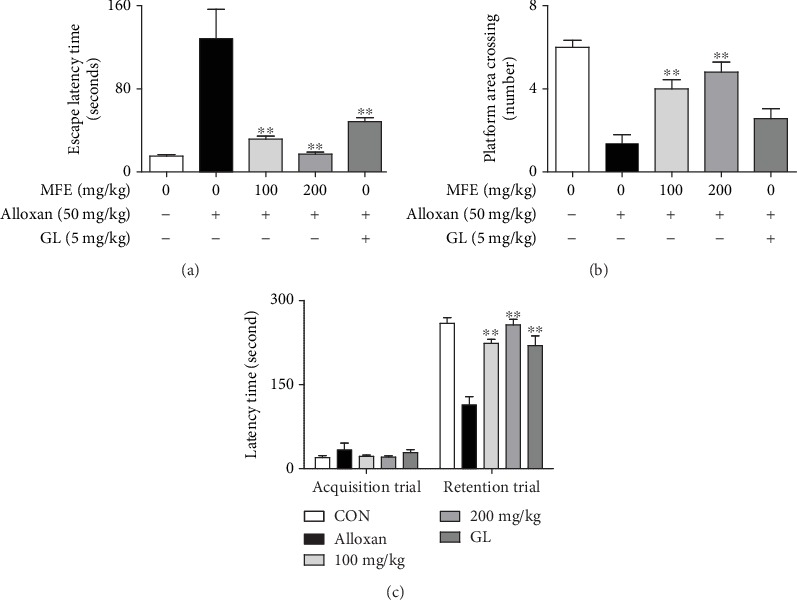
Inhibitory effects of MFE on memory impairment in alloxan-treated mice. Diabetic mice were tested in the Morris water maze and passive avoidance test (acquisition trial) at 11 and 12 weeks, respectively, after MFE or glibenclamide was orally administered. Data are expressed as the mean ± SEM. ^∗∗^*p* < 0.01 versus the alloxan-treated group. (a) Escape latency time. (b) Number of platform area crossing. (c) Latency time.

**Figure 6:**
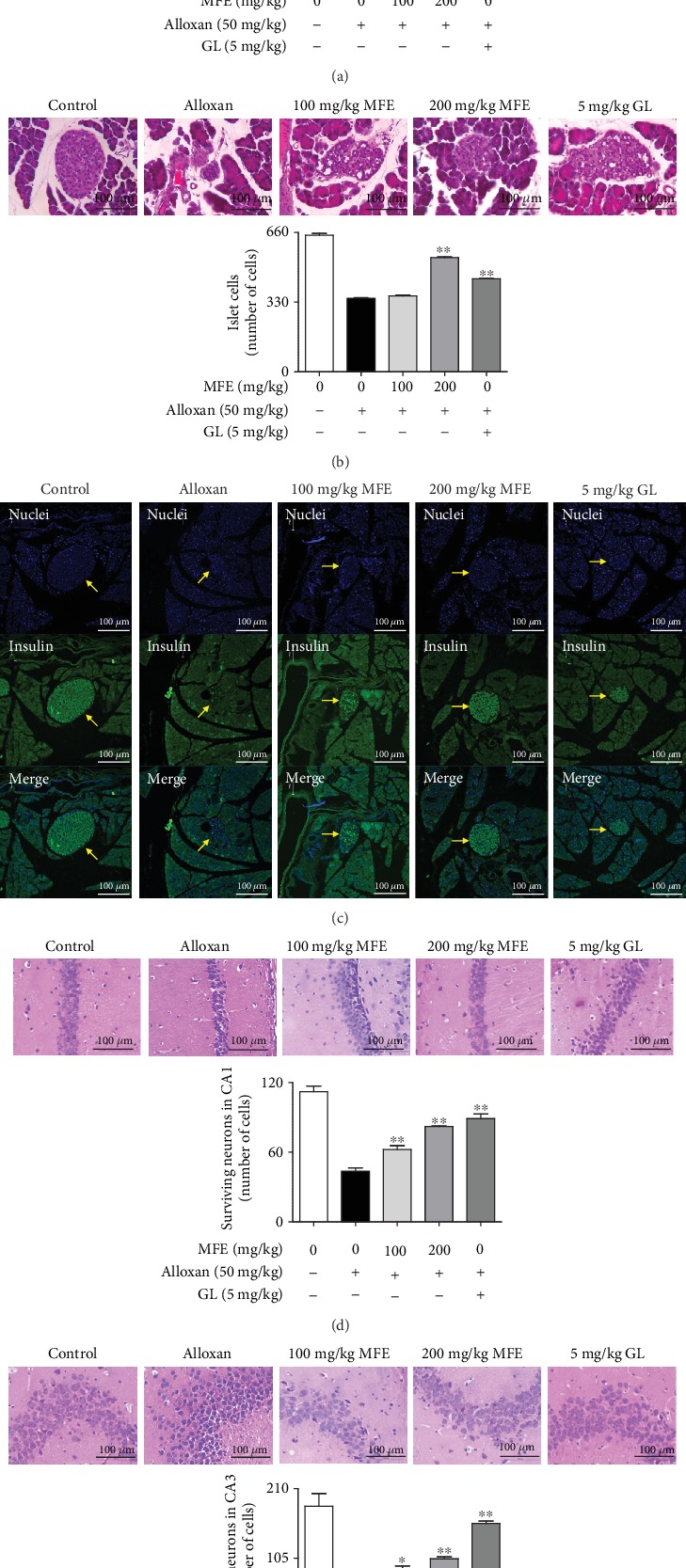
Effects of MFE on histological changes in the kidney, pancreas, and brain. Kidney, pancreas, and brain tissues isolated from the sacrificed mice were fixed. Experimental proceedings are described in the Materials and Methods section. Data are expressed as the mean ± SEM. ^∗^*p* < 0.05 and ^∗∗^*p* < 0.01 versus alloxan-treated group. Arrows indicate the Langerhans islets. (a) Kidney. (b, c) Pancreas. (d) CA1 region. (e) CA3 region.

Following the demonstration of the antidiabetic and antioxidant effects of MFE in alloxan-induced diabetic mice, behavioral tests were conducted to assess its effects on learning and cognition. Histological changes in the kidneys, pancreas, and brain following MFE treatment were analyzed. MFE not only suppressed the escape latency time (Figure 5(a)) but also enhanced the number of platform (Figure 5(b)) and latency time (Figure 5(c)) in alloxan-treated mice. Histological analyses revealed that MFE significantly increased the number of glomerular and islet cells in the kidneys (Figure 6(a)) and pancreas (Figure 6(b)), respectively. Neuronal cells were increased in the CA1 and CA3 regions of the brain (Figures 6(d) and 6(e)). Moreover, MFE increased the expression of insulin in islet cells (Figure 6(c)). These results suggest that MFE directly protects the organization cells of the kidneys, pancreas, and brain against hyperglycemia-induced oxidative stress. These MFE effects might contribute to therapy and prevention of diabetic complications. In particular, protective effects of MFE on beta cells of the pancreas are closely associated with the antidiabetic actions of MFE, potentially suggesting that insulin-dependent type 2 diabetes is curable.

### 3.4. MFE Effects on Expression of APP, P-Tau, BDNF, and P-CREB in Brain Tissue

**Figure 7:**
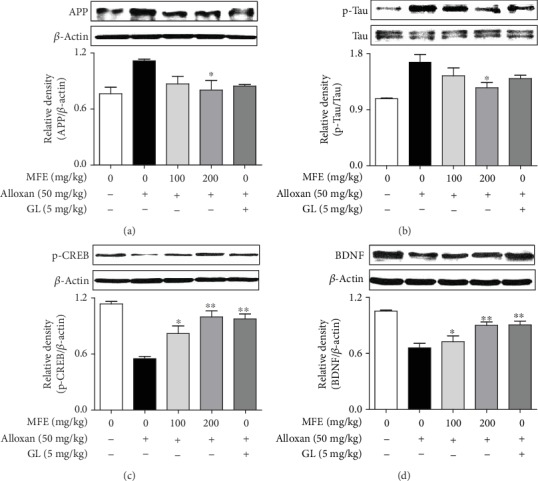
Effects of MFE on APP, p-Tau, BDNF, and p-CREB expression in brain tissue. Experimental proceedings are described in Figure 4. APP, p-Tau, BDNF, and p-CREB expressions were determined. Similar results were obtained in ten independent experiments. ^∗^*p* < 0.05 and ^∗∗^*p* < 0.01 versus the alloxan-treated group. (a) APP. (b) p-Tau. (c) p-CREB. (d) BDNF.

Finally, to elucidate the mechanism of the neuroprotective actions of MFE, the expression of APP, p-Tau, BDNF, and p-CREB in the brain tissue of alloxan-induced diabetic mice was investigated. Previous reports have demonstrated that diabetic memory dysfunction is associated with the upregulation of amyloid-*β* and p-Tau in diabetic rats [27], and BDNF/p-CREB pathway activation is correlated with neuroprotective action against oxidative stress [23, 28]. As shown in Figure 7, MFE not only inhibited APP and p-Tau expression but also increased BDNF and p-CREB expression in the brain tissues of alloxan-treated mice. These findings suggest that MFE regulates both the activation of the p-CREB/BDNF pathway and expression of the Alzheimer-related markers, APP and Tau, in diabetic dementia. Taken together, these results suggest that MFE contributes to reduction in diabetic complications, such as diabetic dementia and renal failure, by regulating hyperglycemia in diabetes. The observed effects of MFE may inform the use of functional food and phytomedicine for diabetes and diabetic complication therapy in clinical populations.

## 4. Discussion

Mulberry fruit has long been used in a food component and traditional herbal medicine in East Asia. Its reported diverse benefits include antiallergic [13], antidiabetic [14], and antioxidant [14] effects. These beneficial effects have been associated with various bioactive components of mulberry fruit, such as polysaccharide [29] and polyphenols [12, 13]. Nevertheless, effects of mulberry fruit on diabetic complications remain to be unreported.

The present study demonstrates that MFE may have beneficial effects on diabetic complications, such as renal failure and Alzheimer's disease, by maintaining blood glucose homeostasis in alloxan-induced diabetic mice. The effects of MFE are associated with antioxidant and antidiabetic activity, such as hypoglycemia and hypolipidemia. In particular, MFE promoted insulin production in the pancreas by protecting beta cells against alloxan-induced ROS. The antioxidant action of MFE was associated with increased antioxidant enzyme activity in the liver, kidneys, and brain. These results indicate that MFE may reduce diabetic complications by protecting the organs against oxidative stress. This conclusion is supported by the finding that MFE significantly reduced alloxan-induced hyperglycemia, hyperlipidemia, glycated hemoglobin, and impaired glucose tolerance in mice. In addition, MFE increased insulin levels and expression in serum and pancreatic tissues, respectively. MFE also reduced increased liver and kidney weight as well as hepatotoxicity (AST and ALT) and nephrotoxicity (UA, BUN, creatinine, and CRP) biomarkers induced by alloxan. MFE not only suppressed lipid peroxidation but also enhanced the antioxidant enzyme activity of SOD and GR in the liver, kidneys, and brain. MFE also recovered the number of glomerular and neuronal cells in the kidneys and hippocampal region, respectively. In behavior tests, MFE inhibited hyperglycemia-induced memory impairment in alloxan-treated mice. In addition, MFE decreased AChE activity, which breaks down acetylcholine [30], and increased ChAT activity, which biosynthesizes acetylcholine [31], in brain tissue. These results suggest that MFE is able to protect beta cells in Langerhans islets against oxidative stress induced by alloxan, which generates ROS in the cells [32]. Consequently, blood glucose homeostasis is maintained by stabilizing insulin secretion and expression in the pancreas of alloxan-induced diabetic mice. Further, the antidiabetic actions of MFE might alleviate diabetic complications, such as diabetic dementia, renal failure, and liver disease. Therefore, the potential therapeutic actions of MFE are closely associated with its antidiabetic and antioxidant activities. Interestingly, MFE at 200 mg/kg showed superior effects than glibenclamide (5 mg/kg) in mostly all results in this study. It suggests that MFE may have a possibility as an antidiabetic herbal drug.

The potential mechanism of the antidiabetic actions of MFE may be associated with bioactive components of the mulberry fruit. Mulberry fruit contains various bioactive components, such as polysaccharide [29], total phenols [12, 13], and flavonoids [13]. In particular, phenolic compounds, including flavonoids, possess antiallergic [13], antidiabetic [14], and neuroprotective [12] bioactivities. A previous study has shown that MFE, similar to mulberry fruit, includes numerous flavonoids and phenolic compounds [11]. Although it has been reported that mulberry fruit polysaccharide has hypoglycemic action, it is extracted with aqueous solution [33]. In addition, water-soluble compounds, such as sugars, are mainly included in the aqueous layer but are insoluble in the ethyl acetate used in the solvent extraction method. Therefore, the antidiabetic actions of MFE are more likely to be associated with various phenolic compounds.

The possible mechanism of antidiabetic complication effects of MFE on the central nerve system might be associated with amyloid-*β* peptide accumulation and activation of the p-CREB/BDNF pathway. Alzheimer's disease pathology is related to the formation of amyloid-*β* peptide plaques and Tau tangles in the brain [7]. In addition, inactivation of insulin signaling in the brain leads to neurofibrillary degeneration by production of Tau tangles [34]. In contrast, activation of the p-CREB/BDNF pathway in the brain leads to survival and proliferation of neuronal cells [23]. Therefore, regulation of the expression of APP, p-Tau, p-CREB, and BDNF in neuronal cells is a key factor for the treatment of diabetic dementia. The current data support this conclusion by showing that MFE not only inhibited APP and p-Tau expression but also activated BDNF and p-CREB expression in the brain tissue of alloxan-treated mice. In addition, MFE reduced oxidative stress and AChE activity, increased ChAT activity and the number of neuronal cells in the CA1 and CA3 regions of the hippocampus, and improved memory. These results might indicate that potential therapeutic effects of MFE for diabetic dementia are associated with neuronal cell protection through regulating Alzheimer's disease-associated pathogens and activation of the p-CREB/BDNF pathway. MFE mechanisms are also associated with the recovery of insulin secretion and expression in the pancreas. Taken together, these results might indicate the use of MFE as a functional food and herbal drug for diabetes-related disease therapy.

## 5. Conclusion

This study demonstrates that MFE probably alleviates diabetes-related complications, such as diabetic dementia, in alloxan-induced diabetic mice. The beneficial effects of MFE included antioxidant, hypoglycemic, and hypolipidemic activities, as well as protection of the pancreas, liver, kidneys, and brain. These findings reveal a potentially novel use for MFE in diabetes-related diseases. Antidiabetic mechanisms of MFE may be associated with its antioxidant and anti-inflammatory activities resulting from rich phenolic compounds, such as phenolic acids and flavonoids. The observed effects of MFE may inform the use of mulberry fruit as a functional food and phytomedicine for diabetes-related disease therapy and prevention. Further cell-based studies are necessary to elucidate how MFE regulates the expression of APP, p-Tau, BDNF, and p-CREB in neuronal cells. Phytochemical studies are required to identify the active compounds that exert neuroprotective action for the development of treatments for diabetic dementia.

## Data Availability

The data used to support the findings of this study are available from the corresponding author upon request.

## Abbreviations

AChE: Acetylcholinesterase
ALT: Alanine aminotransferase
AST: Aspartate aminotransferase
BDNF: Brain-derived neurotrophic factor
BUN: Blood urea nitrogen
ChAT: Choline acetyltransferase
CREB: cAMP response element-binding protein
CRP: Creatinine and C-reactive protein
GR: Glutathione reductase
HbA1c: Hemoglobin A1c
MFE: Ethyl acetate fraction of ethanolic extract of mulberry fruit
ROS: Reactive oxygen species
SOD: Superoxide dismutase
UA: Uric acid.
